# Prolonged venous bleeding due to traditional treatment with leech bite: a case report

**DOI:** 10.1186/1752-1947-5-172

**Published:** 2011-05-06

**Authors:** Bulent Kaya, Orhan Bat, Nuriye Esen Bulut, Hasan Altun, Kemal Memisoglu

**Affiliations:** 1 Fatih Sultan Mehmet Training And Research Hospital, Department of General Surgery, Atasehir, Istanbul, Turkey

## Abstract

**Introduction:**

The medicinal leech, *Hirudo medicinalis*, has been used in the treatment of many diseases for thousands of years. In Turkey, it is used most commonly in the management of venous diseases of lower extremities.

**Case presentation:**

A 25-year-old Turkish woman presented to our emergency room with bleeding from her left leg. She had been treated for varicose veins in her lower extremities with leeches about 24 hours before admission to the emergency room. The bleeding was controlled by applying pressure with sterile gauze upon the wound, and she was discharged. She returned after four hours having started bleeding again. Hemostasis was achieved by vein ligation under local anesthesia.

**Conclusions:**

Leech bite should be evaluated as a special injury. Prolonged bleeding can be seen after leech bites. In such cases, hemostasis either with local pressure or ligation of the bleeding vessel is mandatory.

## Introduction

Leeches are bloodsucking worms usually found in places with fresh water. There are two species of therapeutic leeches, *Hirudo medicinalis *(European medical leech) and *Hirudo michaelseni. H. medicinalis *is about 10 cm in length and 2 g in weight. They have been used for the treatment of many diseases as far back as 2,500 years ago. Headaches, hemorrhoids, and mental illness are some of the disorders that have been treated with leeches.

Treatment with leeches is referred to as hirudotheraphy in modern medicine. Today, medicinal leech therapy is mainly used in plastic surgery and reconstructive surgery for tissue flap salvage [1]. Medicinal leech therapy in Turkey is a traditional treatment for venous disorders of the lower extremities. Most patients get symptomatic relief with this treatment. Leeches are placed onto the lower extremities and they suck the accumulated blood from the dilated veins.

Here, we report the case of a patient with prolonged bleeding after medicinal leech bites. We were able to control the bleeding in our patient by ligation of the punctured, dilated veins with an operation under local anesthesia. We believe that this is the first case report in the English literature concerning prolonged bleeding after leech bite in the treatment of venous disease of the lower extremities.

## Case presentation

**Figure 1:**
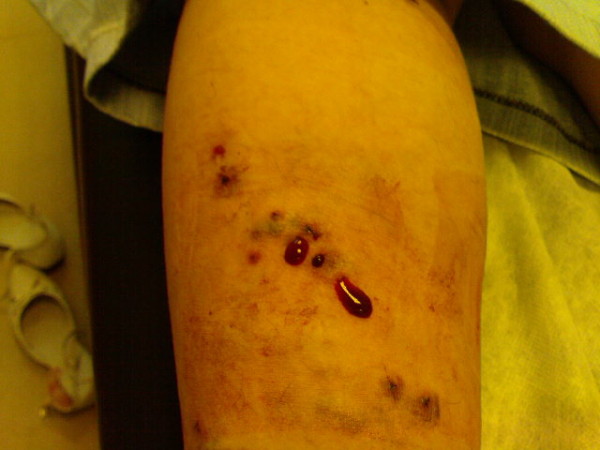
**Bleeding after leech bite**.

A 25-year-old Turkish woman presented to the emergency department with bleeding from her left leg. She had been treated with leeches for varicose veins in her lower extremities about 24 hours prior to her admission. The leeches had stayed in place for three to four hours in the posterior region of her legs. Her medical history was otherwise unremarkable. There was no bleeding diathesis and she was not taking any medication. On physical examination, she was a healthy woman with no findings of distress. Her vital signs were normal. There was oozing bleeding from her left leg. Punctured skin and bleeding, dilated veins were detected (Figure 1). There was no ecchymosis, swelling, or erythema. The bleeding was controlled by compression applied with sterile gauze, and our patient was subsequently discharged.

**Figure 2:**
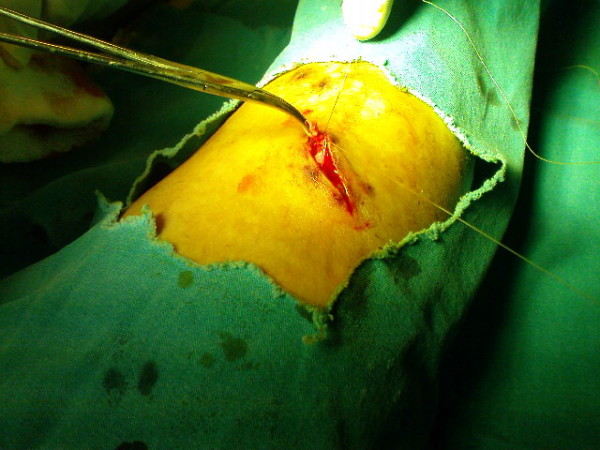
**Ligation of bleeding vessels performed in the emergency room**.

After four hours, our patient was again admitted to our emergency department with recurrent bleeding. The wound was cleaned with antiseptic solution. On physical examination, there were dilated veins with bleeding. The bleeding was active. Her varicose veins were explored under local anesthesia. The veins punctured by leech bite were dissected by operation. They were ligated with 4/0 Vicril Rapide sutures (Figure 2).

After completing hemostasis, the skin was sutured with 3/0 prolene. Our patient was examined seven days after her operation. The wound was clear without any complication.

## Discussion

*H. medicinalis *has two suckers, one in its anterior and one in its posterior region. They usually feed via the anterior suckers in a process that lasts about 20 to 40 minutes. They can suck 10-15 ml of blood and may increase their body size eight to 11 times.

Leeches have different chemical agents within their bodies that are released when salivating (Table 1). Hirudin is a proteolytic inhibitor that has an antagonistic effect to thrombin. The major action of thrombin is the conversion of fibrinogen into fibrin, which is a critical event in the coagulation process. Hirudin, by its inhibitory effect, causes a decrease in platelet aggregation. It is thought that the prolongation of bleeding after leech bite is mainly due to hirudin. Histamine-like substance is another protein that is found in the salivary cells of leeches. It causes vasodilation of the blood vessels. It increases the amount of blood sucked by a leech. Another enzyme, hyaluronidase, facilitates the breakdown of connective tissue by disturbing hyaluronic acid. Beside these chemical agents, Munro *et al. *[2] described another substance found in the saliva of leeches called calin. It has a powerful action as an anticoagulant and mainly inhibits platelet aggregation. The persistent bleeding is likely the effect of the enzymes found in the saliva of leeches. Sustained bleeding may persist as long as seven days. Our patient had uncontrolled bleeding for about 18 hours.

**Table 1:** Chemical substances produced by leeches

Substance	Effect
Hirudin	Anti-thrombotic effect

Histamine-like substance	Vasodilation

Hyaluronidase	Breakdown of connective tissue.

Leeches are commonly used in Turkey in medical treatment of venous congestion of the lower extremities. A diagnosis of leech bite can be made easily. Patients usually have a history of medicinal leech therapy. If a leech is found at the bite site, it can be removed with the use of table salt, vinegar or lignocaine solution.

Bleeding after leech bite in different parts of the body has been reported [3-6]. Leech bite may be associated with morbidity such as serious bleeding and skin infection. Anemia can often be seen with leech infestation. Erysipelas and skin abscess with *Mycobacterium marinum *may also be seen in patients with leech bite. Skin wounds may heal with scar formation. *Aeromonas hydrophila *is a bacterium that can live with leeches symbiotically. It can cause infection after leech bite. Antibiotic prophylaxis in medicinal leech treatment may be recommended. Although it is rare, leech bite may also cause death [7].

Prolonged bleeding after leech bite should be treated seriously. Some bleeding may require transfusion due to loss of large amounts of blood. There are several methods to treat prolonged bleeding after leech bite. Pressure with sterile gauze on the wound is the simplest method. In cases of sustained bleeding, sterile gauze soaked in a thrombin solution can be applied. Desmopressin (1-deamino-8-D-arginine vasopressin; DDAVP) has been reported as an effective agent in controlling bleeding in rats after hirudin infusion [8]. Our patient was treated by applying pressure with sterile gauze upon the wound on her first admission. The bleeding stopped. The patient was admitted again to the emergency room with recurrent bleeding after four hours. Hemostasis was achieved by vein ligation under local anesthesia.

## Conclusions

Leech bite can cause prolonged bleeding. It may even result in death due to blood loss. Leech bite should be evaluated as a special injury with the risk of prolonged bleeding.

## Consent

Written informed consent was obtained from the patient for publication of this case report and any accompanying images. A copy of the written consent is available for review by the Editor-in-Chief of this journal.

## Competing interests

The authors declare that they have no competing interests.

## Authors' contributions

BK performed the surgery. BK, NEB, OB, HA, KM analyzed and interpreted the clinical data, and BK was a major contributor to writing the manuscript. All authors read and approved the final version of the manuscript.
